# A national facility for biological cryo-electron microscopy

**DOI:** 10.1107/S1399004714025280

**Published:** 2015-01-01

**Authors:** Helen R. Saibil, Kay Grünewald, David I. Stuart

**Affiliations:** a Crystallography, Institute for Structural and Molecular Biology, Birkbeck College, Malet Street, London WC1E 7HX, England; b Division of Structural Biology, Wellcome Trust Centre for Human Genetics, University of Oxford, Oxford OX3 7BN, England; c Diamond Light Source, Didcot OX11 0DE, England

**Keywords:** three-dimensional electron microscopy

## Abstract

Three-dimensional electron microscopy is an enormously powerful tool for structural biologists. It is now able to provide an understanding of the molecular machinery of cells, disease processes and the actions of pathogenic organisms from atomic detail through to the cellular context. However, cutting-edge research in this field requires very substantial resources for equipment, infrastructure and expertise. Here, a brief overview is provided of the plans for a UK national three-dimensional electron-microscopy facility for integrated structural biology to enable internationally leading research on the machinery of life. State-of-the-art equipment operated with expert support will be provided, optimized for both atomic-level single-particle analysis of purified macromolecules and complexes and for tomography of cell sections. The access to and organization of the facility will be modelled on the highly successful macromolecular crystallography (MX) synchrotron beamlines, and will be embedded at the Diamond Light Source, facilitating the development of user-friendly workflows providing near-real-time experimental feedback.

## Abstract 2

This review provides a brief update on the use of cryo-electron microscopy for integrated structural biology, along with an overview of the plans for the UK national facility for electron microscopy being built at the Diamond synchrotron.

## 1. Introduction   

**Figure 1:**
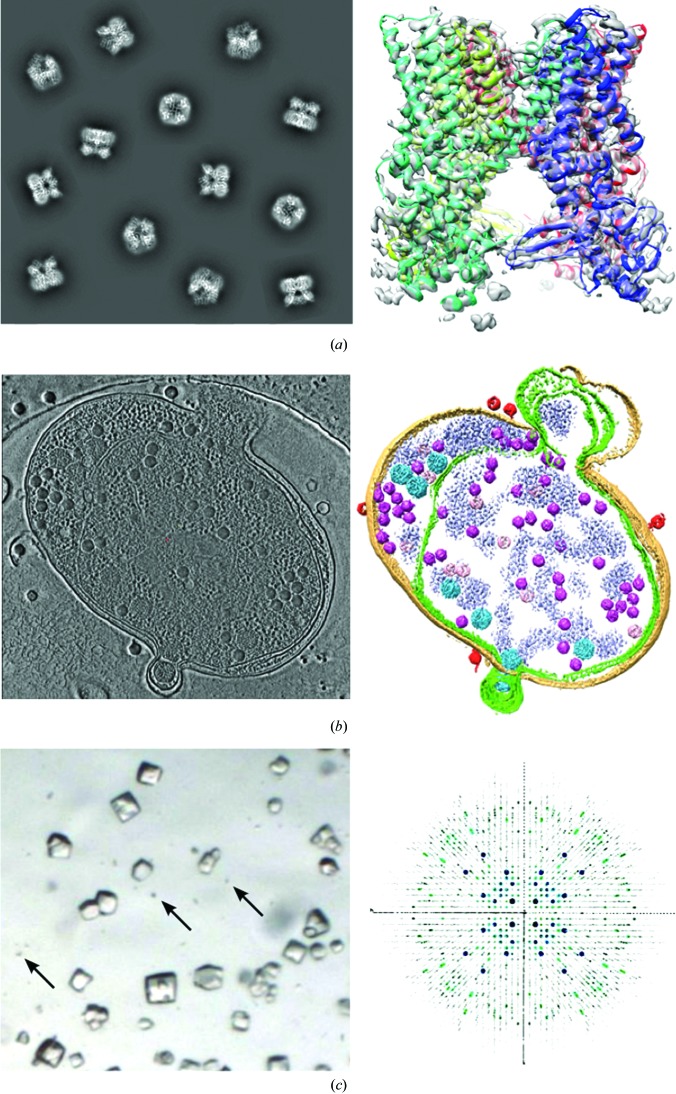
Overview of current methods in biological cryo-EM. (*a*) Single-particle reconstruction of the TrpV channel (Liao *et al.*, 2013 ▶; EMD-5778). Left, projected views representing idealized single-particle data. Right, three-dimensional reconstruction of the mainly α-helical tetramer, coloured by subunit, with the fitted secondary structure. (*b*) Zernicke phase-contrast cryo-ET of a virus-infected cyanobacterial cell. Left, section through the tomogram; right, segmented view of the cell with the viruses in pink. Reproduced by permission from Macmillan Publishers Ltd, Dai *et al.* (2013 ▶), copyright (2013). (*c*) Electron diffraction of lysozyme microcrystals. Left, optical micrograph of small crystals with microcrystals indicated by arrows. Right, representation of the three-dimensional electron diffraction data. Figures reproduced or modified from Shi *et al.* (2013 ▶) under a Creative Commons Attribution license.

From the origins of electron microscopy (EM) the method has made major contributions to biology, but the recent technical and methodological developments outlined below have expanded both the scope and the precision of the method, so that cryo-transmission EM is now a central pillar of structural biology (Henderson, 2004 ▶; Frank, 2006 ▶; Cheng & Walz, 2009 ▶; Lučič *et al.*, 2013 ▶). It covers a wide range of sample size and resolution from tomographic analysis of sections of whole cells at 20–40 Å resolution down to single-particle analysis of less than 200 kDa molecular mass at around 3 Å resolution (see Fig. 1 ▶ for an overview). Furthermore, it is a powerful tool not only for detailed structure determination but also for the characterization and quality control of assemblies being expressed and purified for structural analysis. In this context, we envisage the national facility fitting in by providing access at the top end of an increasingly broad use of the method. The national facility will work to encourage growth in the EM community by providing training programmes to generate an expanding, more confident and more demanding user base. In particular, we expect a broadening of the user community to encompass structural and cell biologists who are not EM specialists but are attracted by the potential of the method. This will markedly increase the demand for top-end equipment, just as high-end MX beamlines have enabled progress on some of the most biologically significant but challenging crystallographic problems, empowering a user base that now has very high expectations of facilities.

Transmission electron microscopy of vitrified samples was developed over 30 years ago (Dubochet *et al.*, 1988 ▶). In recent years, the capabilities of single-particle EM and electron tomography (ET) have expanded dramatically, benefitting from hardware and software advances, notably stable and coherent electron sources (field emission guns), stable specimen stages (including multi-sample cartridges), automation and high-speed direct electron detectors, along with powerful statistical methods for reconstruction and sorting out heterogeneity in data sets (Jensen, 2010*a* ▶,*b* ▶,*c* ▶; Suloway *et al.*, 2005 ▶; Ruskin *et al.*, 2013 ▶; Li *et al.*, 2013 ▶; McMullan *et al.*, 2014 ▶; Scheres, 2012 ▶; Orlova & Saibil, 2011 ▶). Further developments, *e.g.* phase plates and aberration correction, are ongoing (Dai *et al.*, 2013 ▶; Wang *et al.*, 2011 ▶). However, the continuing hardware developments are increasingly putting state-of-the-art facilities for this type of work out of the reach of most laboratories. It is becoming prohibitively expensive to acquire and run high-end cryo-EMs and electron detectors, and the demands on engineering expertise, high-tech infrastructure and building services strain the capabilities of most universities and small institutes. Furthermore, the logistical problems of providing a safe, functioning 24/7 facility are more easily solved in a central facility operating with support staff available day and night. For this reason, the UK structural biology community has prioritized the establishment of a central facility for high-end EM.

## 2. Methods   

Work on the structural biology of macromolecular machinery is now moving beyond the operation of individual proteins to encompass whole systems. Illustrations of the power of this complex approach come, for example, from virus, proteasome, ribosome and membrane-inserted structures (Li *et al.*, 2013 ▶; Liao *et al.*, 2013 ▶; Zhang *et al.*, 2010 ▶; Amunts *et al.*, 2014 ▶; Fig. 1 ▶). Until now, such high-resolution studies have been performed on highly purified samples, but it is now clear that the next major challenge is to obtain the same level of understanding in the context of cells and tissues (Al-Amoudi *et al.*, 2007 ▶; Lučič *et al.*, 2013 ▶). To achieve this integration of molecular and cellular information will require significant further development of the methods of correlative microscopy (Faas *et al.*, 2013 ▶) and multiscale imaging (Russel *et al.*, 2012 ▶). We will briefly summarize the major methods of electron imaging, which use transmission microscopy applied to biological specimens cryo-preserved in vitreous ice.

### 2.1. Single-particle analysis   

This method is suitable for purified complexes that are large enough to give sufficient signal for detection and alignment (Frank, 2002 ▶; van Heel *et al.*, 2000 ▶). In practice, the lower limit has until recently been about 200 kDa, although in theory 100 kDa should be achievable and we expect further progress towards this (Henderson, 2004 ▶). In contrast there is no upper mass limit for single particles, but the sample thickness must be well under 1 µm for TEM to avoid limitations caused by multiple scattering of electrons passing through the specimen and other electron optical problems. The principle is simple: a single projection image is obtained from each of a large number of similar particles. If the particles are in random orientations then combining the information from the different particles will enable a full three-dimensional reconstruction of the average structure. This process is very computationally intensive compared with crystallography since the process of crystallization precisely defines the relative position and orientation of all of the particles in the crystal, whereas in cryo-EM single-particle analysis these parameters must be determined for each particle using an iterative computational procedure. With a state-of-the-art system, the resolution obtainable from suitable single-particle samples is often limited by the order and homogeneity of the sample, with atomic detail resolvable in the best cases. In practice, the criteria for good samples are usually similar for EM and X-ray crystallography and so the availability of ‘crystallization grade’ material is an excellent starting point for a single-particle analysis (although the concentration required for EM analysis is usually considerably less than that required for crystallization). It is important to note that, aside from sample considerations, biological structure determination by cryo-EM is strictly limited by the inherently low signal-to-noise ratio and by radiation damage to the specimen. This contributes to the requirement of at least tens of thousands of asymmetric units for a high-resolution reconstruction. Therefore, improvements in signal contrast in cutting-edge systems (*e.g.* phase plates), increased stability in the microscope and, most notably, increased speed and detective quantum efficiency of direct electron detectors provide major advantages. Movie-mode acquisition with direct detectors enables correction for beam-induced motion by realignment of sub­frames, leading to a marked increase in the efficiency of the method at higher resolution (Li *et al.*, 2013 ▶). With sufficiently large and rigid biological assemblies (Zhang *et al.*, 2010 ▶) and direct detectors it is becoming routine to determine a single-particle structure in atomic detail (at 3–4 Å resolution) using state-of-the-art equipment (Amunts *et al.*, 2014 ▶; Li *et al.*, 2013 ▶; Liao *et al.*, 2013 ▶; Fig. 1 ▶ *a*). With a growing number of analyses resulting in resolutions better than 4 Å, there is increasing crossover in computational methods for structure analysis and refinement between X-ray crystallography and electron microscopy. Thus, the leading X-ray refinement programs, *PHENIX* and *REFMAC* (Moriarty *et al.*, 2014 ▶; Murshudov *et al.*, 2011 ▶), are both now capable of performing model refinement against electron-microscopy maps. There is still scope for improvement in the methods, with relatively few published ‘fully refined’ EM structures so far (Amunts *et al.*, 2014 ▶). Moreover, there are still problems to solve in methods for validation and resolution determination (Chen *et al.*, 2013 ▶).

#### 2.1.1. Computational sorting to resolve dynamics from multiple snapshots   

**Figure 2:**
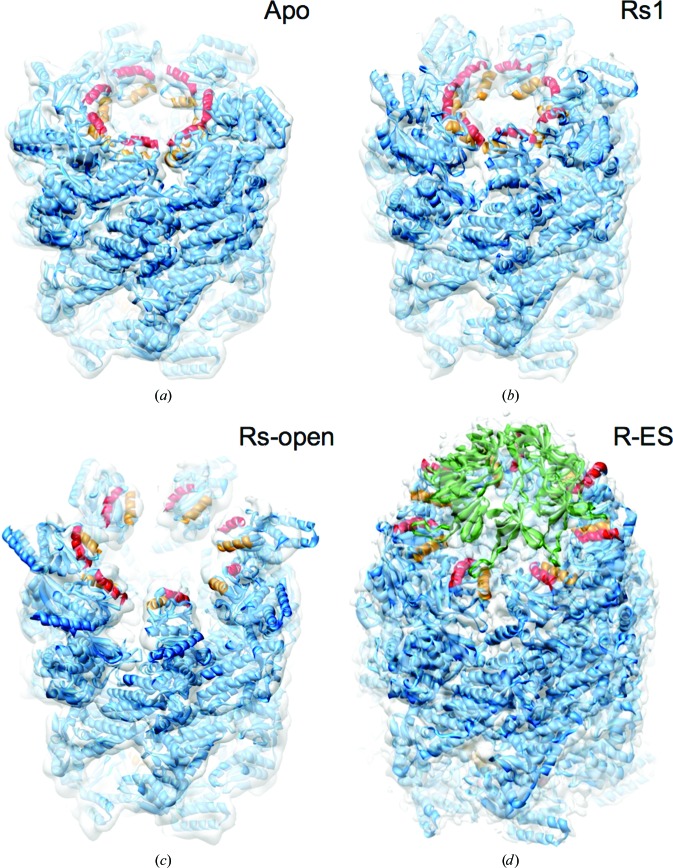
Dynamics of GroEL–ATP (Clare *et al.*, 2012 ▶). Cryo-EM maps (transparent surfaces) and flexible fitting show some of the main structural states determined by multivariate statistical analysis of a 60 000-particle data set. Helices H and I, which denote the hydrophobic binding sites for non-native proteins, are shown in orange/red and the GroES lid is shown in green. (*a*) Apo GroEL, (*b*) GroEL–ATP_7_, Rs1 state, (*c*) GroEL–ATP_7_, Rs-open state, (*d*) GroEL–GroES–ATP.

Software developments now make it possible to extract multiple high-quality structures from data sets of heterogeneous complexes in different functional states (Clare *et al.*, 2009 ▶, 2012 ▶; Fischer *et al.*, 2010 ▶; Fig. 2 ▶), but resolution is often limited by the throughput of data collection and processing. Depending on how many conformations are present and how much they differ, various statistical methods can be used either in two dimensions or three dimensions to detect and separate the different conformations. If an accurate separation is achieved, then the resolution of each structure will be determined by the number of asymmetric units and the conformational homogeneity/rigidity of that subset. For more difficult and labile complexes, biochemical approaches such as Grafix, which involves fractionation of complexes in the presence of dilute glutaraldehyde (Kastner *et al.*, 2008 ▶), can be used to stabilize transient assemblies. These approaches provide a series of snapshots that can provide fundamental insight into the dynamics of macromolecular machines carrying out key activities such as protein synthesis or folding, and can map major conformational changes (Clare *et al.*, 2012 ▶; Fischer *et al.*, 2010 ▶; Zhang *et al.*, 2008 ▶; Scheres, 2012 ▶).

### 2.2. Electron cryo-tomography (cryo-ET)   

Tomography provides lower resolution information but allows a full three-dimensional reconstruction of a single object by recording a series of views (Mastronarde, 2005 ▶). The method provides mechanistic details of nanoscale cellular processes such as cargo transport, virus uncoating, membrane modulation and membrane trafficking (Lučič *et al.*, 2013 ▶; Dai *et al.*, 2013 ▶). It reveals a new landscape of previously unknown details of cell structure, enabling the analysis of macromolecular complexes functioning in their native environment (Al-Amoudi *et al.*, 2007 ▶; Fig. 1 ▶ *b*). In addition, the method provides ideal tools to reveal new information on host–pathogen interactions at the supramolecular level (Zeev-Ben-Mordehai *et al.*, 2014 ▶).

A major limitation is that only the thinnest (<<1 µm) regions of cells can be studied intact. However, specimen thinning by cryo sectioning or ion beam milling relieves this restriction, giving access to all parts of the cell and enabling the study of cellular processes that take place, for example, in and around the nucleus. Vitreous sectioning using a diamond knife is relatively cheap and gives access to any cell or tissue regions that can be prepared by high-pressure freezing, which is suitable for specimens around 100 µm thick and up to a few millimetres in diameter (see, for example, Al-Amoudi *et al.*, 2007 ▶). The main limitation of this approach is mechanical damage during sectioning, which causes variable mechanical compression and crevassing, limiting the useful section thickness to ∼50 nm. This is an insufficient depth of view for large assemblies such as nuclear pores. In the alternative technique of ion beam milling, the cryo-sample is mounted in a scanning EM and a beam of heavy ions is used to ablate the surface to produce a lamella. This method has fundamental advantages, since it reduces the problems of mechanical damage and can produce 200–400 nm thick lamellae of the sample (Marko *et al.*, 2006 ▶; Rigort *et al.*, 2012 ▶). However, ion beam milling is not yet routine and requires expensive specialist equipment, and issues of locating objects of interest within the specimen and capturing them in a lamella harbouring these objects have not yet been solved.

For cryo-ET the conflicting requirements of low electron dose to minimize radiation damage and higher dose for sufficient signal to noise provide the major limitations for three-dimensional reconstruction. A full tilt series must be collected of each object, with repeated electron exposures delivering a high cumulative dose, and each tilt exposure must have sufficient signal for alignment. Although these requirements limit cryo-ET to nanometre resolution, it is playing an increasingly important role in defining cellular complexes and in extending structural biology from the molecular level to the cellular level. Furthermore, recent advances in electron detection, and likely further improvements, are having a major impact on the effectiveness of electron tomography by providing more recorded signal for a given electron dose.

### 2.3. Sub-tomogram averaging   

It is possible to extract sub­regions from tomograms for alignment, classification and averaging, establishing a methodological continuum between tomography and single-particle analyses (Grünewald *et al.*, 2003 ▶; Bartesaghi *et al.*, 2012 ▶) and providing intermediate resolution. Structures that cannot be isolated intact but that are present as multiple copies in intact systems are computationally extracted from tomograms and treated as three-dimensional single particles. Alignment, classification and averaging of such tomogram subregions increases their signal-to-noise level and combines different orientations, leading to significant improvements in resolution (Schur *et al.*, 2013 ▶). This can reveal molecular-level three-dimensional structures of previously inaccessible cellular assemblies, and has reached subnanometre resolution in favourable situations. This method is still at an early stage of development and is likely to be applicable to many important biological questions.

### 2.4. Correlative microscopy   

**Figure 3:**
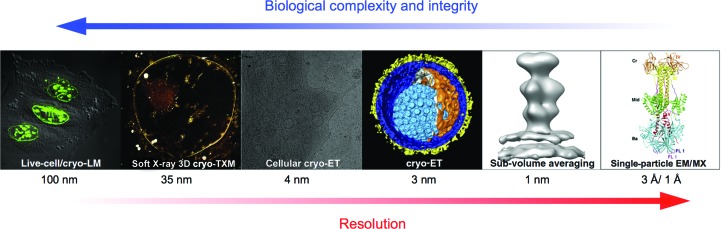
Integrated structural biology approach combining correlative light microscopy, soft X-ray cryo-microscopy and electron cryo-microscopy with high-resolution structure information, thus enabling the dissection of dynamic processes at different levels of resolution and complexity. The biological process is visualized by light microscopy (LM), transmission X-ray cryo-microscopy (cryo-TXM), electron cryo-tomography (cryo-ET), single-particle cryo-EM and/or macromolecular crystallography (MX). Adapted from Zeev-Ben-Mordehai *et al.* (2014 ▶).

In order to determine the molecular structures of cellular assemblies, a useful approach is to image the same structures with both cryo-ET and fluorescence microscopy (Fig. 3 ▶). The rapidly expanding power of fluorescence microscopy is used to identify areas and events of interest within cells, which are then examined in molecular detail by electron imaging. Correlative microscopy is well established for plastic-embedded EM samples, but fluorescence cryo-imaging is still in its infancy, with limited commercial equipment and no commercial cryo-immersion objectives, thus rendering it hard to fully exploit the developing panoply of super-resolution light microscopy methods. Nevertheless, various correlative studies, including the first super-resolution imaging of flash-frozen live cells, have been reported (van Driel *et al.*, 2009 ▶; Hagen *et al.*, 2012 ▶; Chang *et al.*, 2014 ▶; Perkovic *et al.*, 2014 ▶; Kaufmann *et al.*, 2014 ▶). Furthermore, the complete correlative chain requires live cell imaging, rapid cryo-preservation at a selected time, sample thinning and cryo-fluorescent imaging followed by EM. To obtain time and space correlation throughout this chain remains a matter of active development. The same methodology is also required for the full exploitation of soft X-ray microscopy, which will be offered at beamline B24 of Diamond. This emerging imaging area builds on sample-preparation procedures originally established for cryo-EM and therefore fits well into the correlative pipeline (Hagen *et al.*, 2012 ▶).

### 2.5. Electron crystallography   

Electron microscopes configured for single-particle analysis and tomography can also be used for recording electron diffraction. An important early development in three-dimensional EM was electron crystallography of two-dimensional crystals, particularly of membrane proteins (Henderson & Unwin, 1975 ▶). Two-dimensional crystals provide extended rods of diffraction perpendicular to the plane of the crystals. To obtain the three-dimensional structure, data must be combined from many such crystals recorded at different tilt angles, thereby sampling different positions along the diffraction rods. In addition to electron diffraction, images of the crystals are recorded to allow correction of lattice disorder by using correlation methods to search for the actual locations of unit cells. In practice, this approach has had limited application, primarily because of the difficulty of producing sufficiently large and well ordered two-dimensional crystals. However, a common byproduct of attempts to grow three-dimensional crystals for MX are microcrystals. If they are less than ∼500 nm thick, and can be vitrified in a thin layer on an EM grid, they can be analysed by electron crystallography. In a recent application of this approach (microED), a series of electron diffraction patterns, each at very low electron dose, is recorded during continuous tilt of the sample stage (Nannenga *et al.*, 2014 ▶). Combining diffraction data from a few crystals with different orientations on the EM grid provided a complete data set enabling structure determination at 2.9 Å resolution (Shi *et al.*, 2013 ▶; Fig. 1 ▶ *c*). Currently, phasing of such data is performed by molecular replacement. This is potentially a significant development for challenging crystallo­graphic problems.

## 3. Implementation   

The intention is to offer access to and some training in all of the above techniques at the national EM facility at Diamond. Here, we will briefly describe the model for the facility and then indicate the implementation plan.

### 3.1. The macromolecular crystallography model   

There are many common features between MX and cryo-EM, and the EM field is following a similar trajectory to the early development of MX. Although EM does not require a centralized radiation source like a synchrotron, the high-end equipment has cost and infrastructure requirements that are very similar to those of a synchrotron beamline, as well as complex software that would benefit from coordinated curation and adoption of international standards. The plan is therefore to construct a high-end EM facility according to the beamline model, taking advantage of the existing scientific and technical infrastructure and user-programme organization at a synchrotron.

Structural biology in the UK has a long history of the use of synchrotron radiation for MX, with specialized beamlines from the outset at the world’s first dedicated synchrotron light source, the SRS at Daresbury, which was commissioned in 1981 (Duke & Johnson, 2010 ▶). This early focus on MX developments has helped, over a period of more than 30 years, to coordinate hardware and software efforts across the whole field, extending to an involvement in the international community. The construction of the Diamond synchrotron, initiated ten years ago, has reinvigorated UK activity in this area. The development of MX provides an excellent model for how centralized resources and coordination can move a whole scientific field forward. For example, software coordination led by the Collaborative Computational Project CCP4 has transformed disparate MX software into a coherent, robust commercial-quality package with a huge international impact (http://www.ccp4.ac.uk/). It has consolidated data structures, facilitated data exchange and acted as a framework into which innovative algorithms can be embedded. In addition, it has facilitated the broader uptake of the method and its use by industry.

In the beamline model, a team of dedicated scientists supported by specialist teams covering engineering, controls, vacuum, data acquisition and analysis software operating a user-focused facility aims to provide data of the highest quality as quickly and easily as possible and to provide the software tools to enable, as far as possible, close to real-time first-cut data analysis. This fits the needs of a national EM facility and provides opportunities for synergism and coordination in software development and sample preparation with the soft X-ray microscopy also being developed at Diamond (beamline B24; http://www.diamond.ac.uk/Beamlines/Imaging/B24.html).

At this stage, the lack of standards and consistent user interfaces represents a barrier to the effective use of the method by nonspecialists. To address these problems and to accelerate progress, a CCP-EM equivalent of CCP4 has been launched (http://www.ccpem.ac.uk/). CCP-EM coordinates with international efforts through the EM Data Bank (EMDB) based at the European Bioinformatics Institute (http://www.emdatabank.org/). The EMDB plays a key role in the development and maturation of the field through public deposition of three-dimensional EM data and coordination of international efforts to set up standardized validation tools (Henderson *et al.*, 2012 ▶) and integration of cellular and molecular structure-determination techniques and data archiving (Patwardhan *et al.*, 2014 ▶).

### 3.2. Microscopes   

The main function of the Diamond facility is to provide two state-of-the-art 300 kV cryo-microscopes with stable, cartridge-type sample holders and the latest generation of electron detectors, incorporating correction for beam-induced motion, as well as phase plates. Integration with potential future developments such as aberration correctors is envisaged, if and when desirable. At least one of the systems will include an energy filter, which is important for electron tomography. In addition a lower specification, lower voltage machine will be available for triaging of samples and for research carried out at the facility.

### 3.3. Sample-preparation equipment   

Sample-preparation equipment will be available for both molecular and cellular samples and will include a vitrification robot, access to a high-pressure freezer, cryo-sectioning and a dual-beam cryo-SEM which will allow the preparation of thin lamellae from frozen cellular samples on grids by focused ion beam milling.

### 3.4. Biological containment   

The national facility will provide category 2 containment resources for single-particle analysis, cryo-tomography, correlative fluorescence/EM and automated data collection, with additional access to higher containment-level facilities through the Oxford Particle Imaging Centre (OPIC) at the Wellcome Trust Centre for Human Genetics, University of Oxford for contained cryo-EM and tomography of infectious particles (http://www.opic.ox.ac.uk). OPIC has committed to provide 20% of its user programme through the peer-review system for the national facility on the same basis as the machines based at Diamond. It is equipped with a stable 300 kV microscope with post-column energy filter and direct electron detector.

### 3.5. Automated data collection   

High-end electron microscopes can be operated remotely, as is the case with MX. It is possible to collect a substantial amount of data from each sample grid, especially for single-particle analysis. The improved electron optical and mechanical stability in high-end systems is also important in addressing the problem that EM has traditionally been a low-throughput, manually operated technique by facilitating continuous, unattended data collection (Suloway *et al.*, 2005 ▶). In particular, automation enables the collection of very large data sets, which are essential for the determination of high-resolution structures of flexible complexes. In a stable cryo-microscope with a low rate of sample contamination, fully automated data collection requires the software to recognize and select areas with suitable ice thickness and can run for one to several days given a suitable grid. This is generally performed on nanofabricated grids with regular arrays of holes in the support film (Quispe *et al.*, 2007 ▶). In semi-automated data collection, the user first selects regions for data collection, which are then queued up for automated recording. We anticipate that full automation in EM, as in MX, will require further development before it can be applied reliably to the majority of samples. Tomograms can also be collected at a set of specified positions. A typical experiment might involve several days of data collection from a few grids to generate hundreds of thousands of particles. Conversely, for rigid complexes this improved efficiency means that data-collection times are reduced, especially since direct electron detectors dramatically reduce the number of particles that are required for a high-resolution analysis.

### 3.6. High-throughput data management and analysis   

The data rate from a single high-end microscope with the current generation of direct electron detectors is between one and a few terabytes per day. This is at least as much as a current MX beamline at Diamond, so that the structures that have been put in place at Diamond for data management, including data archiving, will be required. To this end, the national EM facility will integrate directly with the centralized high-performance file systems and compute clusters that have been established at Diamond, which automatically pass data to an off-site system for archiving. The demands of EM activity will necessitate a significant enhancement of the present systems to provide pipelines for real-time EM data analysis. Indeed, this problem has already been addressed by some in the EM field; for instance, Appion is a web-based user interface that controls a sequence of operations for automated particle picking, contrast transfer-function correction, alignment, classification and Euler angle determination (Lander *et al.*, 2009 ▶). For a well behaved sample such a pipeline would start operating on data as it comes off the microscope and could generate initial class averages, three-dimensional maps or tomograms on the fly during data collection. The analogous pipelines developed for MX at Diamond now routinely and automatically process user data within a few minutes of data collection (Winter & McAuley, 2011 ▶). Using the MX experience, the National Facility will aim to give real-time feedback to the EM user about, for example, the number of particles found, the data quality and the initial tomograms. The computational support groups at Diamond are already engaged with this problem, and our expectation is that an initial pipeline will be in place when the facility at Diamond takes its first users. Establishing a complete pipeline will be facilitated by the presence of the CCP-EM group adjacent to the Diamond synchrotron. The full extent of the computing needs is hard to estimate, since whilst hardware becomes faster, software becomes more sophisticated. However, we expect that a current state-of-the-art 1000-core cluster would more or less keep up with the first-pass analysis needs of a single high-end EM. Thus, the extent to which Diamond will be able to offer full downstream analysis of the data remains an open question because of the much greater computational demands. However, a properly run facility for remote data analysis would clearly be a great enabler of uptake of the method amongst new user communities.

### 3.7. User access   

The goal is to create a national laboratory for state-of-the-art cryo-EM and to build a fully operational user facility within five years. This facility will run 24 hours a day and our target is to offer a user programme of ∼200 days a year on each high-end machine, with peer-validated access *via* an independent panel, very similar to a synchrotron beamline, and run through the Diamond User Office. The user programme will be based on the six-month Diamond beamtime application systems for application, sample logging and report gathering. It is envisaged that access will be provided in similar ways to those presently offered to MX users, with the major route being a beamline allocation group (BAG) system, allowing a group of principal investigators, typically representing an institution, to manage a joint allocation, and an additional rapid access route to provide some flexibility.

### 3.8. Training and outreach to new user communities   

The success of a centralized national facility will depend on its usability for the structural, molecular and cell biology communities. The methods for both cutting-edge electron microscopy and electron tomography are highly specialized and require considerable training and expertise, even more so once data are collected. In order to extract useful results from the image data, images must be processed using software which is complex, often lacking intuitive user interfaces and documentation, and in some cases commercial and very expensive. The EM laboratory will include training facilities and devote some staff time to guiding visiting users in image processing in close cooperation with CCP-EM (Wood *et al.*, 2015 ▶). The EMDB also has training and outreach, and we will jointly aim to provide this to both structural and cell biologists who wish to make use of EM methods.

### 3.9. Management and metrics for success   

Top-level governance will be by a Programme Board, with independent scientific scrutiny to be provided by a Scientific Advisory Committee. The staff model will be broadly parallel to that for a Diamond beamline, with a Director, Senior Facility Scientist, Facility Support Scientist and PDRA, with Research Assistants providing high-level support for training and sample preparation.

We will judge success by metrics including publications, depositions in the EMDB/PDB, methods development, training, user-group development and cooperation between user groups nationally and internationally. In the first instance we do not anticipate significant commercial use of the facility, but we will explore working with industry to encourage their uptake of the emerging methods.

## 4. Conclusions   

This is a very exciting time for three-dimensional EM at all levels. The field has seen dramatic progress from ‘primitive blobology’ to cutting-edge structural and cellular biology. It covers much of the resolution range relevant to biology and now extends to the point where it can produce results of value to medicinal chemists. In overall direction it is following some decades behind MX, so that the trajectory is to some extent predictable. The development of direct electron detectors has been a key factor in the recent surge in the power of cryo-EM. However, the high costs and the need for specialist expertise and major infrastructure for data collection and processing mean that it is becoming imperative to have centrally funded national facilities equivalent to synchrotrons for MX.
